# PROS1/AXL signaling protects mice from lethal influenza infection by inducing M2 macrophage polarization: The PROS1/AXL axis protects mice from lethal influenza infection

**DOI:** 10.3724/abbs.2025169

**Published:** 2025-10-29

**Authors:** Wenbo Zhu, Shao Wang, Shuangquan Liu, Qiang Fu, Hongbo Zhang

**Affiliations:** 1 The First Affiliated Hospital Clinical Medical Research Center Hengyang Medical School University of South China Hengyang 421001 China; 2 Institute of Animal Husbandry and Veterinary Medicine Fujian Academy of Agriculture Science Fuzhou 350013 China; 3 The First Affiliated Hospital Clinical Laboratory Hengyang Medical School University of South China Hengyang 421001 China; 4 The First Affiliated Hospital Department of Rheumatology and Immunology Hengyang Medical School University of South China Hengyang 421001 China; 5 Department of Pediatrics University of Pittsburgh School of Medicine Pittsburgh PA 15224 USA

**Keywords:** PROS1, AXL, macrophage, polarization, influenza infection

## Abstract

AXL, a member of the TAM (Tyro3, AXL, and Mertk) subfamily of RTKs, is abundantly expressed in lung tissue and has been implicated in viral infections and lung injury. PROS1, one of the ligands known to activate AXL, functions as an immunomodulator in many diseases. However, the role of PROS1/AXL signaling in influenza A virus (IAV) infection and infection-induced lung injury is largely unknown. In this study, we find that the exogenous administration of PROS1 mitigates lung injury and protects mice from lethal infection by IAVs through the activation of AXL. PROS1 induces the phosphorylation of AXL, which in turn recruits Gab1 and p85, a regulatory subunit of PI3K, to form a complex that activates Gab1 and its downstream PI3K/AKT/mTOR in alveolar macrophages. *Gab1* knockdown *in vivo*, or LY294002 (a PI3K inhibitor), abolishes the PROS1/AXL-induced protective activity against lethal influenza infection in mice. We also show that PROS1/AXL signaling induces M2 polarization of alveolar macrophages through Gab1 activation both *in vitro* and *in vivo*. *Gab1* knockdown inhibits M2 macrophage accumulation in IAV-infected lungs and attenuates the protective effect of PROS1. These results indicate that PROS1/AXL signaling can activate Gab1 in macrophages and induce macrophage polarization to an anti-inflammatory M2 phenotype, thereby eliciting protective activity against lethal infection with IAVs. These data also highlight the PROS1/AXL signal as a novel therapeutic target for IAV infection.

## Introduction

Influenza A viruses (IAVs) are zoonotic, negative-sense, single-stranded RNA viruses that are closely associated with worldwide pandemics [1]. Annual outbreaks of IAV cause considerable morbidity and mortality in communities [2]. The deadliest of these outbreaks, the 1918 pandemic “Spanish Flu”, caused by an unusually virulent H1N1 subtype of IAV, resulted in 500 million infections and 50 million deaths [3]. In recent years, avian influenza variants, including H5N1 and H7N9, have been able to cross-infect humans and have high rates of infection and lethality compared with other IAV strains, with pandemic potential risk [4]. Severe influenza infections are often lethal, manifesting as cytokine storms, inflammation, flooding of alveolar spaces, and even acute respiratory distress syndrome and respiratory failure. However, the factors that control IAV replication and limit lethal lung inflammation remain largely unknown. Vaccines and anti-influenza medications, such as neuraminidase inhibitors, are available but may be much less effective in immunocompromised populations or those infected with drug-resistant IAVs [ 5, 6] . Therefore, the identification of novel antiinfluenza targets and agents is urgently needed.

Tyro3, AXL, and Mertk (TAM) receptors are a subfamily of receptor tyrosine kinases (RTKs) that are expressed in various cells and tissues. In the lung, TAM receptors are expressed in alveolar type II epithelial cells (ATII cells), alveolar macrophages and neutrophils [ 7– 10] . Vitamin K-dependent protein growth arrest specific protein 6 (Gas6) and protein S (PROS1) are two ligands that bind to and activate the TAM receptor [11]. TAM receptors and their ligands have potent immunomodulatory functions. Currently, the most studied TAM receptors (Tyro3/AXL/Mertk) recognize phosphatidylserine on apoptotic cells via the bridging molecules Gas6 and PROS1, thus mediating the clearance of apoptotic cells [ 12, 13] . TAM receptor activation-mediated efferocytosis and inhibition of inflammatory pathways protect the brain from ischemic stroke and viral infections [ 14, 15] . However, the role of the immunomodulatory functions of TAM receptors and their ligands in combating IAV infection and infection-induced lung pathology is unclear. Exogenous administration of PROS1 or Gas6 alleviated acute lung injury in mice by inhibiting inflammatory cytokine secretion through the activation of AXL [ 16, 17] . AXL plays a crucial role in inducing protective antiviral adaptive immunity by limiting the immunosuppressive effects of type I IFNs [18]. Alveolar macrophages (AMs) are critical for immunity to influenza A virus (IAV) infection [19], and PROS1 has been reported to promote an immunosuppressive microenvironment in gliomas by polarizing tumor-associated macrophages toward the M2 phenotype [20], suggesting that PROS1 may also affect alveolar macrophage polarization and function. All of the above studies suggest an important and beneficial role for the TAM receptor and its ligands in counteracting IAV. However, contradictory findings suggest that AXL contributes to the development of lung pathology after viral infection [10]. Therefore, an in-depth investigation of the role and intrinsic immunoregulatory mechanisms of TAM receptors and their ligands in lethal IAV infection and infection-induced lung injury is necessary.

In this study, we identified a novel function of PROS1/AXL signaling in the induction of anti-inflammatory M2 macrophage polarization through the activation of Gab1 and downstream PI3K/AKT/mTOR, thereby protecting mice from lethal IAV infection. These findings suggest that PROS1/AXL signaling could be a novel therapeutic target for influenza infection by promoting M2 macrophage polarization.

## Materials and Methods

### Animals and chemicals

Wild-type mice and AXL ^–/–^ mice (cat. 005777; Jackson Laboratories, Bar Harbor, USA), all on the C57BL/6J background and aged 5–8 weeks (18–22 g), were used. All the mice were housed in a specific pathogen-free laboratory and maintained on a standard 12 h light/12 h dark cycle. All experimental procedures carried out in this study were approved by the Animal Care and Use Committee of the University of Pittsburgh. All chemical drugs and antibodies used are listed in Supplementary Table S1.

### Virus preparation and virus titer determination

Viruses were prepared as previously described [21]. Influenza A virus/Puerto Rico/8/1934 (IAV/PR8), influenza A virus/New York/1682/09 (IAV/NY1682) and influenza A virus/California 04/09 (IAV/CA04) (all influenza virus strains used in this study are stored in the –80°C refrigerator in our laboratory) were grown in the chorioallantoic fluid of 11-day-old chicken embryos, purified on a discontinuous sucrose gradient, dialyzed against PBS to remove sucrose, and finally aliquoted and stored at –80°C until needed.

Viral titers were determined in Madin-Darby canine kidney (MDCK) cells and expressed as IFU/mL, which was defined as the number of cells positive for the NP signal per 1 mL of virus sample [22]. Briefly, MDCK monolayers inoculated with diluted viral samples were fixed, permeabilized, immunostained with an anti-NP monoclonal antibody and an HRP-labeled secondary antibody, and finally developed with TrueBlue Peroxidase Substrate (50-78-02; SeraCare, Milford, USA).

### Intranasal infection of IAVs

Five-week-old mice were anesthetized with ketamine (K2753; Sigma, St Louis, USA) and inoculated intranasally with IAVs from different viral titers, 15 μL per nasal cavity (30 μL, total). After infection, the mice were continuously monitored for survival, weight loss and clinical signs for up to 14 days. Infected and drug-treated lungs were surgically resected and used to detect titers of virus infection, pathological changes in the lungs, and alterations in molecular signaling pathways.

### Drug administration

For mouse treatment, recombinant Gas6 (200 μg/mL, 10 μL) or rPROS1 (200 μg/mL, 10 μL) was injected intranasally at 2 μg/day/per mouse from day 1 to day 6 after IAV infection. LY294002 (80 μg/50 μL in saline with 0.1% DMSO) was also inoculated intranasally at 80 μg/day/per mouse from day 1 to day 6 after IAV infection. The group challenged with saline containing 0.1% DMSO was used as a vehicle control. Gab1 siRNA or control siRNA (1 nmol/μL, 10 μL, SR419225; OriGene, Rockville, USA) was administered intranasally or injected via the trachea 48 h before IAV infection via TransIT-TKO transfection reagents (10 μL/per mouse, MIR2155; Mirusbio, Madison, USA), as previously reported [23].

For cell treatment, rPROS1 (stock 20 μg/mL, 10 μL per dose, in 1 mL medium/well; final working concentration: 0.2 μg/mL) was added to the supernatant of isolated alveolar macrophages (AMs) and incubated for the indicated time. Control siRNA or Gab1 siRNA was transfected into isolated alveolar macrophages by using Lipofectamine RNAiMAX (13778030; Invitrogen, Carlsbad, USA) 48 h before rPROS1 treatment.

### BALF collection and lung tissue homogenization

After the mice were euthanized, the thoracic cavity was surgically exposed, the trachea was incised, and the lungs were lavaged with 2 mL of sterile cold PBS. Approximately 2 mL of bronchoalveolar lavage fluid (BALF) was collected per mouse, centrifuged at 700 *g* for 10 min at 4°C and stored at –80°C for further analysis. The entire right lung was collected and gently homogenized in 1 mL of sterile ice-cold PBS containing protease inhibitors (P1010; Beyotime, Shanghai, China) at 4°C. Tissue-free lung homogenates were collected and stored at –80°C for use in real-time PCR. Cell-free lung supernatants lysed with RIPA lysis buffer (P0013B; Beyotime) were collected and stored at –80°C for cytokine detection via ELISA. The left lung was fixed with 10% neutral formaldehyde (HT501640; Sigma) and subsequently subjected to H&E staining.

### Detection of the wet lung/body weight ratio

The body weights of the mice were first measured, and then the upper lobe of the right lung of each mouse was collected and immediately measured as the weight of the wet lung. The ratio of wet lung weight to body weight in the mice was calculated to assess pulmonary edema.

### Isolation of alveolar type II epithelial cells from murine lungs

Murine alveolar type II epithelial cells (AEC2 cells) were isolated according to methods previously reported [ 24, 25] . Briefly, the lungs were surgically exposed, 10 mL of PBS was instilled through the pulmonary artery to remove erythrocytes, followed by 2 mL of dispase (3535235; Corning, Corning, USA) through the trachea and 0.5 mL of 1% low-melting-point agarose (16520050; Life Technologies, Carlsbad, USA) to seal the upper airway. The lungs were excised from the mice and incubated in a 15-mL tube containing 0.5 mL of dispase for 45 min at 25°C before being transferred to a 60-mm tissue culture dish containing 5 mL of HEPES-buffered DMEM (10-013-CV; Corning) with 100 units/mL DNase I (AMPD1-1KT; Sigma Aldrich) for mechanical dissection. The cell suspensions were filtered through 70-μm and 40-μm cell strainers (352350 or 352340; Corning) to obtain single-cell suspensions. The collected cells were subjected to CD45 negative selection via mouse CD45 microbeads (130-052-301; Miltenyi Biotec, Bergisch Gladbach, Germany) and a MidiMACS Separator (130-042-302; Miltenyi Biotec). The harvested CD45 ^–^ cells were subsequently incubated with a biotin-conjugated anti-EpCAM antibody (13-5791-82; eBiosciences, San Diego, USA) at 4°C. After washing, the cells were subsequently incubated with streptavidin-conjugated microbeads (130-048-102; Miltenyi Biotec), and the cell-bead complex was harvested with a MidiMACS Separator. The isolated CD45 ^–^EpCAM ^+^ cells were identified as AEC2s and verified by western blotting with an anti-SP-C antibody.

### Isolation of alveolar macrophages (AMs) and alveolar neutrophils from murine lungs

Murine alveolar macrophages (AMs) and alveolar neutrophils were isolated according to methods previously reported [ 26, 27] . Briefly, the lungs were perfused with 10 mL of ice-cold PBS through the pulmonary artery to flush circulating blood cells. The perfused whitened lungs were dissected, transferred to a sterile petri dish containing 3 mL of DMEM, and chopped into pieces of less than 1 mm. To the lung tissue blocks, 300 μg/ml Liberase TL (5401020001; Roche, Basel, Switzerland) and 5 U/mL DNase I (AMPD1-1KT; Sigma Aldrich) were added, mixed gently with a pipette and incubated for 25 min at 37°C in an incubator. Dissociated lung tissue was filtered through a 70-μm cell strainer (352350; Corning Falcon) and prepared as a single-cell suspension free of erythrocytes. The cells were stained with the following antibodies: anti-F4/80 (756635; BD Biosciences, San Jose, USA), anti-CD11c (565452; BD Biosciences), anti-CD11b (568486; BD Biosciences), and anti-Ly6G (746448; BD Biosciences) and sorted via flow cytometry via a cell sorter (644832; BD Biosciences). The alveolar macrophage (AM) population was designated F4/80 ^+^CD11b ^low^CD11c ^+^ cells. The alveolar neutrophil population was designated as F4/80 ^low^CD11c ^low^CD11b ^high^Ly6G ^high^. The isolated alveolar macrophages and alveolar neutrophils were identified via western blotting via an anti-MGL1/2 antibody (for macrophages) and an anti-MPO antibody (for neutrophils), respectively.

### Real-time PCR

Real-time PCR was performed as previously described [28]. TRIzol reagent (15596018; Invitrogen) was used to extract total RNA from IAV/PR8-infected or uninfected whole lung tissue. The reverse transcription of total RNA was performed with DNase I and a cDNA synthesis kit (K1682; Thermo Scientific, Waltham, USA). The real-time PCR procedure was then performed via TB Green ^TM^ Premix Ex Taq ^TM^ II (RR820A; Takara, Tokyo, Japan) on an ABI-7500 system. Gene amplification with a two-step PCR program was performed as follows: predenaturation at 95°C for 30 s, followed by 40 cycles at 95°C for 15 s and 56°C for 30 s. The relative quantification of all genes was standardized to that of *GAPDH*. The normalized data of each gene were analyzed via the 2 ^–∆Ct^ method. All primers used are listed in Supplementary Table S2.

### Western blot analysis

The cells and lung tissues were collected, homogenized and lysed with RIPA lysis buffer (P0013B; Beyotime). The protein supernatant was obtained by centrifugation, and its concentration was determined with a BCA detection kit (CW0014S; CWBIO, Beijing, China). The total protein sample was denatured by boiling at 95°C in loading buffer for 5 min, subjected to polyacrylamide gel electrophoresis and transferred to PVDF membranes (ISEQ00010; Millipore, Bedford, USA). The membranes were blocked with 5% milk, incubated with the corresponding primary antibodies at 4°C overnight and subsequently incubated with secondary antibodies labeled with HRP at room temperature for 2 h. Signals were visualized via SuperKine ^TM^ supersensitive ECL luminescent solution (BMU102-CN; Abbkin, Wuhan, China). The intensities of the protein bands were quantitatively analyzed via ImageJ software.

### Coimmunoprecipitation (Co-IP) assay

Harvested lung tissue (> 500 mg) from rPROS1-injected mice or vehicle-treated mice (injected with 10% DMSO in saline as a control) was homogenized and lysed with ice-cold RIPA lysis buffer in the presence of protease inhibitors. The protein supernatants were collected by centrifugation and quantified via a BCA kit. First, the protein was precleared by adding 20 μL of Dynabeads protein G (10004D; Thermo Fisher Scientific) to 1 mg of total protein, stirring gently and incubating at 4°C for 2 h. Then, the supernatant was collected by centrifugation at 500 *g* for 10 min and subsequently incubated with IgG or specific antibodies in the presence of Dynabeads protein G overnight at 4°C. The beads-proteins complex was recovered with a magnetic rack, washed with ice-cold RIPA buffer and boiled at 95°C for 5 min in 1× loading buffer to free the bound protein. The samples were subsequently analyzed via western blot analysis. Total protein (20 μg) was used as an input control.

### ELISA

BALF and protein supernatants from cells and homogenized lung tissues were collected for cytokine detection via ELISA. Test procedures were carried out in accordance with the protocols provided by the reagent manufacturer. The optical density of each well was measured with an automatic microplate reader (Filter Max F5; Molecular Devices, Sunnyvale, USA) at 450 nm. The ELISA kits used for the mice in this study are listed in Supplementary Table S1.

### Pathological examination and injury scoring of the lung

The lung tissues were cut into 5-μm-thick sections after being fixed with formaldehyde, dehydrated with ethanol, and embedded in paraffin. These sections were subjected to hematoxylin-eosin (H&E) staining and evaluated for pathological damage to the lungs via microscopic observation. The lung injury score is a composite score derived from the independent assessment of each parameter, including neutrophil infiltration of the alveolar spaces, alveolar septal thickening, pulmonary hemorrhage, and hyaline membrane formation, by two double-blind pathologists [29]. The scores are divided according to the following criteria: a score of 0 is no injury; a score of 1 is mild injury; a score of 2 is moderate injury; and a score of 3 is severe injury.

### Statistical analysis

All values are expressed as the mean ± standard deviation (SD). GraphPad Prism 8 software was used. Survival rates were analyzed via the log-rank (Mantel-Cox) test. Student’s *t*-test for two groups or two-way ANOVA for multiple groups were conducted to analyze significant differences. *P* values less than 0.05 were considered significant.

## Results

### IAV infection increases the expression of Gas6 and PROS1 and their receptor AXL

**Figure 1:**
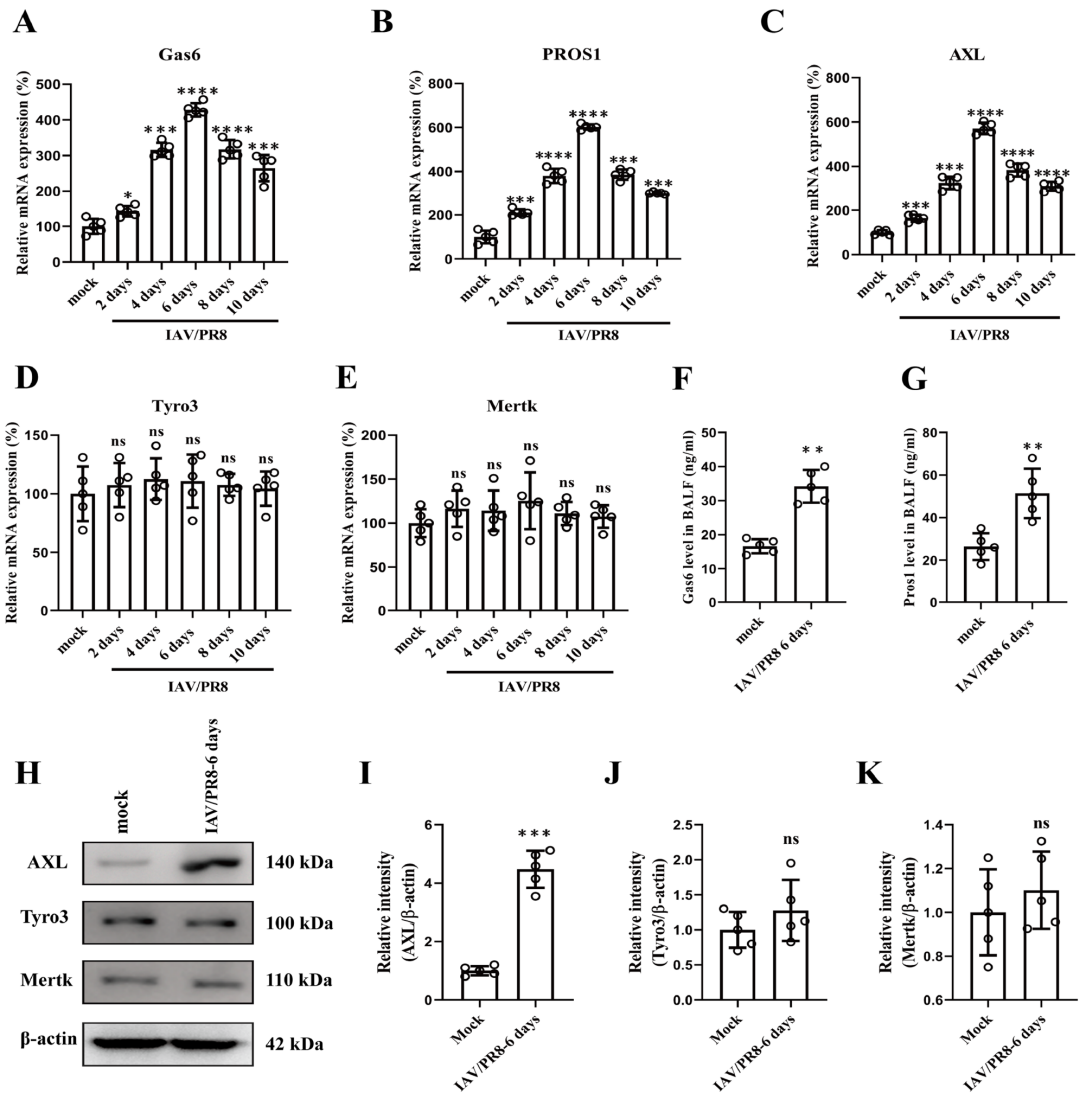
Figure 1 IAV infection increases the expression of Gas6 and PROS1 and their receptor AXL (A–E) Results of quantitative real-time PCR showing the mRNA levels of Gas6,PROS1,AXL,Tyro3, and Mertk in the lungs of the mock group and the groups at 2, 4, 6, 8, and 10 days after IAV/PR8 infection. n = 5 for each group. (F,G) Gas 6 and PROS1 levels in bronchoalveolar lavage fluid (BALF) in the mock and IAV/PR8 infection groups. n = 5 for each group. (H) Western blot analysis of the protein expression of TAM receptors in the lungs of mock- and IAV/PR8-infected mice. n = 5 for each group. (I–K) Statistical charts with densitometric analysis for quantification of the western blot results in (H). t-test for two-group comparisons. *P < 0.05, **P < 0.01, ***P < 0.001 and ****P < 0.0001. ns, not significant.

To investigate the immunoregulatory functions of TAM receptors and their ligands in counteracting IAV infection and infection-induced pulmonary pathology, we first assessed the basal expression of TAM receptors and their ligands in lung tissue and BALF from control mice and IAV/PR8-infected mice. As shown by real-time PCR assay, the mRNA expression levels of *Gas6* and *PROS1* were significantly elevated in lung tissue on day 2 after infection with 100 infectious units (IFU) of the influenza virus strain IAV/PR8. The expression peaked on day 6 and remained elevated until day 10 ( Figure 1A,B). Among the TAM receptors, only the mRNA level of *AXL* increased significantly after 2 days of IAV infection, peaked on day 6, and persisted until day 10, whereas the expression of Tyro3 and Mertk did not differ at any time point ( Figure 1C–E). Similar to the trend in mRNA, ELISA results showed that the secretion of Gas6 and PROS1 was also significantly increased in the BALF on day 6 after IAV infection ( Figure 1F,G). The protein levels of AXL detected by western blot analysis were also significantly elevated on day 6 after infection, whereas the protein expression of Tyro3 and Mertk in the lung tissue was unchanged ( Figure 1H–K).

### Treatment with rPROS1 inhibits IAV/PR8 infection-induced lethality in mice

**Figure 2:**
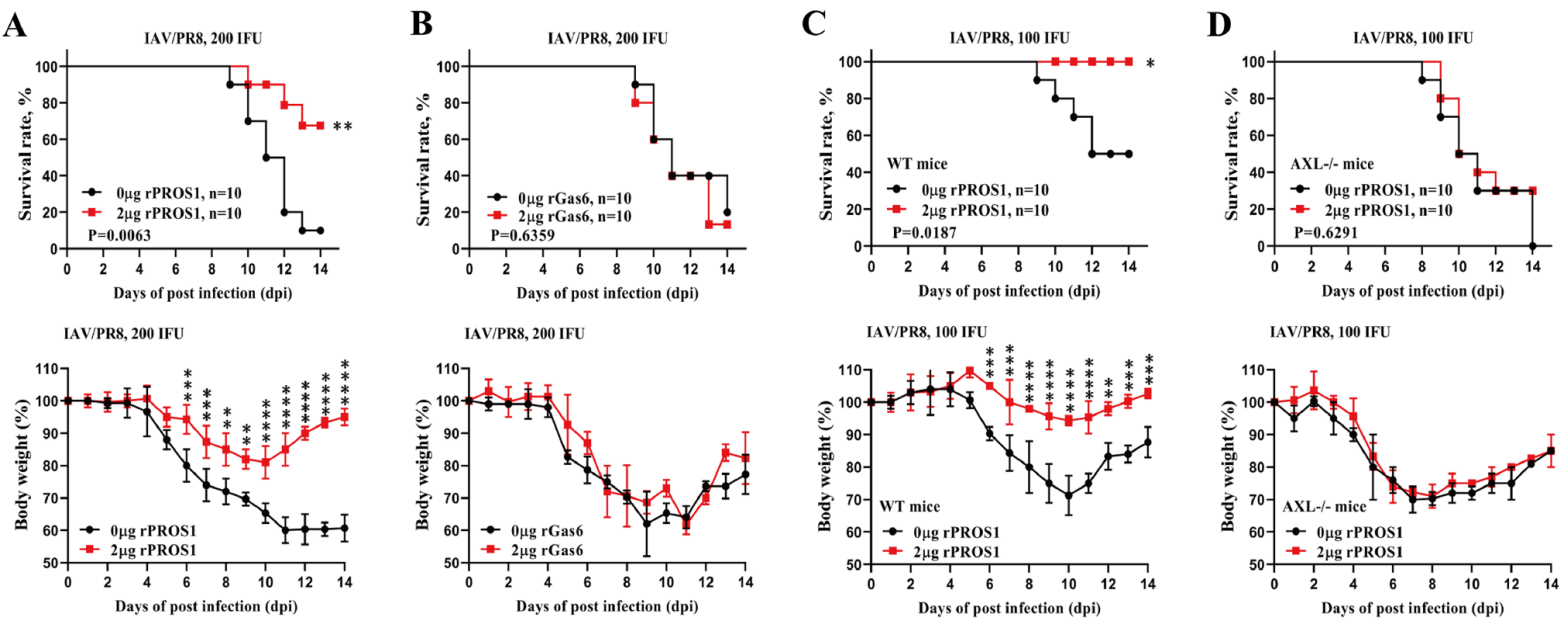
Figure 2 Treatment with rPROS1 inhibits IAV/PR8 infection-induced lethality in mice (A) Survival rate (%, upper panel) and body weight loss (%, lower panel) of WT mice intranasally administered with either the buffer alone (0 μg/mouse) or rPROS1 (2 μg/mouse) from day 1 to day 6 after transnasal infection with 200 IFU of IAV/PR8. The error bars represent the standard deviations (SDs). n = 10 for each group. (B) Survival rate (%, upper panel) and body weight loss (%, lower panel) of WT mice intranasally administered with either the buffer alone (0 μg/mouse) or rGas6 (2 μg/mouse) from day 1 to day 6 after transnasal infection with 200 IFU of IAV/PR8. Error bars, SDs. n = 10 for each group. (C) Survival rate (%, upper panel) and body weight loss (%, lower panel) of WT mice intranasally administered with either the buffer alone (0 μg/mouse) or rPROS1 (2 μg/mouse) from day 1 to day 6 after transnasal infection with 100 IFU of IAV/PR8. Error bars, SDs. n = 10 for each group. (D) Survival rate (%, upper panel) and body weight loss (%, lower panel) of AXL–/– mice intranasally administered with either the buffer alone (0 μg/mouse) or rPROS1 (2 μg/mouse) from day 1 to day 6 after transnasal infection with 100 IFU of IAV/PR8. n = 10 for each group. Error bars, SDs. Survival rates were analyzed via the log-rank (Mantel-Cox) test. Body weight loss was analyzed via two-way ANOVA. *P < 0.05, **P < 0.01, ***P < 0.001 and ****P < 0.0001.

Gas6 and PROS1 attenuate alveolar inflammation and alleviate acute lung injury [ 16, 17] , whereas AXL is required for T-cell priming and antiviral immunity [18]. However, their roles in influenza virus infection have not been reported. Given that the expression of Gas6, PROS1 and AXL was increased during IAV infection, we further explored whether they provide beneficial protective effects during IAV infection. Large doses (compared with self-secreted amounts) of Gas6 or PROS1 (2 μg/per mouse/day) were administered intranasally into wild-type C57BL/6 (WT) mice from day 1 to day 6 after transnasal infection with 200 IFU of IAV/PR8. Survival rate analysis showed that approximately 80%–90% of the control mice, which received only vehicle buffer, died by 14 days post-infection (dpi) ( Figure 2A,B). However, rPROS1, but not rGas6, markedly reduced the mortality of infected mice in a dose-dependent manner ( Figure 2A,B and Supplementary Figure S1). Weight loss was also mitigated in the rPROS1-treated, IAV/PR8-infected mice compared with the control mice ( Figure 2A,B and Supplementary Figure S1). These results indicated that treatment with PROS1 could confer protection against IAV/PR8 infection-induced lethality in mice.

Given the important role of AXL in antiviral immunity, whose expression is also increased during IAV infection, we subsequently investigated whether the protective role of rPROS1 in lethal IAV infection is executed through its receptor AXL. Similarly, we intranasally administered rPROS1 (2 μg/per mouse/day) to AXL ^–/–^ ( *AXL*-knockout mice) and WT mice from day 1 to day 6 after infection with 100 IFU of IAV/PR8. Considering that AXL ^–/–^ mice may be highly susceptible to IAV/PR8 infection, we used lower dose (100 IFU) of IAV/PR8 in this experiment. Survival rate and weight loss analysis showed that rPROS1 consistently reduced the mortality and weight loss of infected WT mice ( Figure 2C). However, the mortality and weight loss of infected AXL ^–/–^ mice were not mitigated by rPROS1 ( Figure 2D). These results confirmed that PROS1 exerts its protective effect against lethal IAV infection by targeting its receptor AXL.

### PROS1/AXL signaling suppresses lung pathology in IAV-infected mice

**Figure 3:**
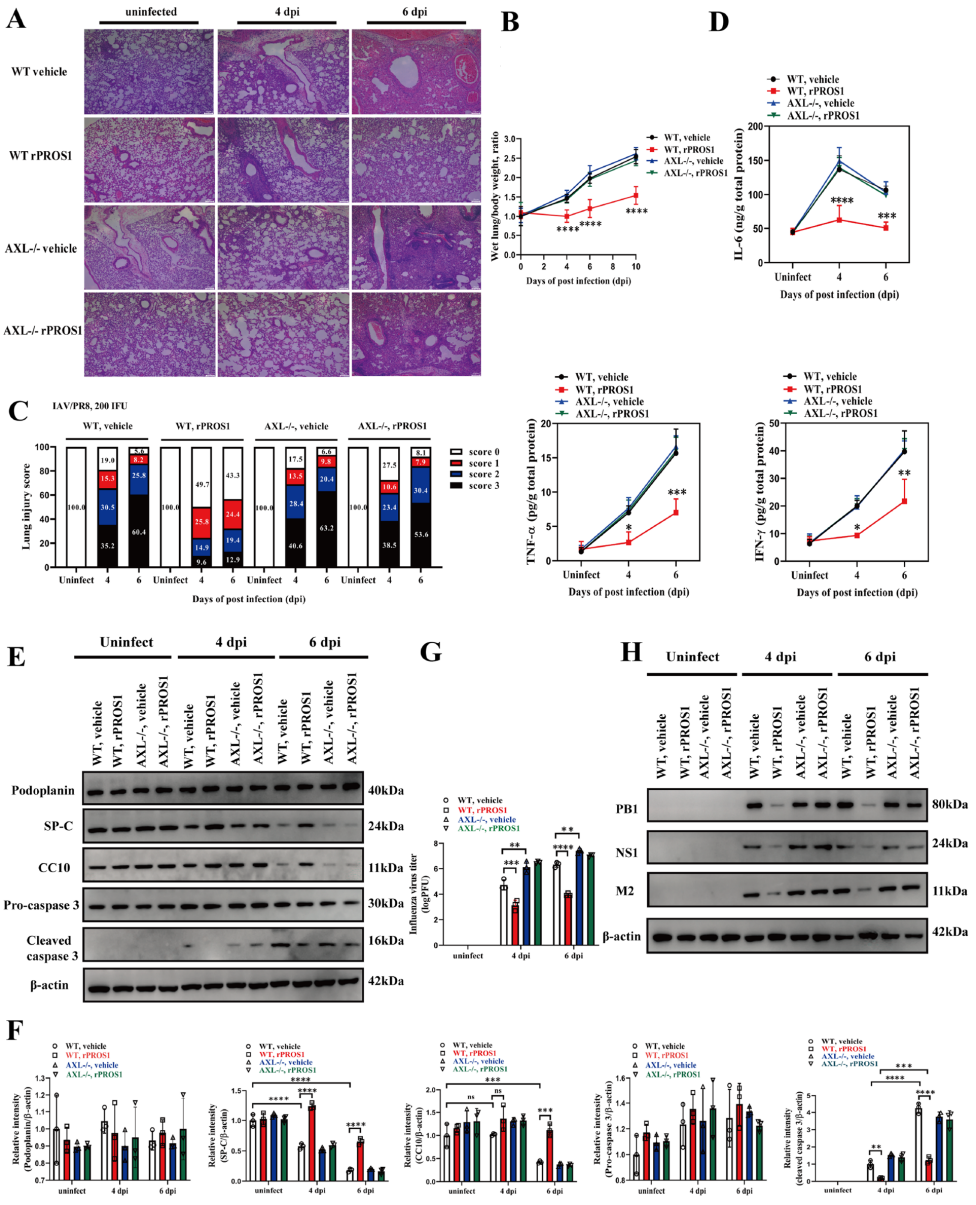
Figure 3 PROS1/AXL signaling suppresses lung pathologies in IAV-infected mice (A) Hemotoxylin-eosin-stained lungs from vehicle buffer- and rPROS1-treated WT or AXL–/– mice uninfected and at 4 and 6 dpi with 200 IFU of IAV/PR8. Scale bar, 25 μm. The ratio of wet lung weight/body weight (B), injury score (C), and levels of proinflammatory cytokines (D) in the lungs of vehicle buffer- and rPROS1-treated WT or AXL–/– mice uninfected and at 4 and 6 dpi with 200 IFU of IAV/PR8. (E) Western blot analysis of the AT1 cell marker podoplanin, the AT2 cell marker SP-C, the Clara cell marker CC10, pro-caspase 3, and cleaved caspase 3 in lungs from vehicle buffer- and rPROS1-treated WT or AXL–/– mice uninfected and at 4 and 6 dpi with 200 IFU of IAV/PR8. (F) Statistical charts with densitometric analysis for quantification of the western blot results in (E). (G) Viral titers in the lungs of vehicle buffer- and rPROS1-treated WT or AXL–/– mice at 4 and 6 dpi with 200 IFU of IAV/PR8. (H) Western blot analysis of the viral proteins PB1, NS1, and M2 in the lungs of vehicle buffer- and rPROS1-treated WT or AXL–/– mice uninfected and at 4 and 6 dpi with 200 IFU of IAV/PR8. n = 3 per group for WB or viral titer assays. n = 10 per group for other assays. The data were analyzed via two-way ANOVA. *P < 0.05, **P < 0.01, ***P < 0.001 and ****P < 0.0001.

To investigate the effects of PROS1/AXL signaling on IAV-infected lung pathologies, we histopathologically (H&E staining) evaluated lung tissue from vehicle- and rPROS1-treated WT or AXL ^–/–^ mice that were either uninfected or infected with IAV/PR8 (200 IFU) for 4 or 6 days. Compared with those in uninfected lungs, infiltration of inflammatory cells, mild hemorrhage and hyaline membrane formation were present in the parenchyma and bronchiolar areas of IAV/PR8-infected lungs and were particularly evident at 6 dpi. In addition, these pathological changes were significantly milder in the lung tissues of the rPROS1-treated IAV/PR8-infected WT mice than in the lung tissues of the vehicle-treated IAV/PR8-infected WT mice at 4 or 6 dpi. Nevertheless, these pathological changes were not ameliorated by rPROS1 treatment in IAV/PR8-infected AXL ^–/–^ mice ( Figure 3A). The wet weights of the lungs of the rPROS1-treated IAV/PR8-infected WT mice were significantly lower than those of the other groups, suggesting that rPROS1 reduces exudates in lung tissues via AXL ( Figure 3B). The lung injury score revealed that rPROS1 treatment significantly reduced the proportion of WT mice but not AXL ^–/–^ mice with scores of “score 2” or “score 3” (representing severe lung tissue injury) ( Figure 3C). ELISA results showed that the levels of the inflammatory cytokines interleukin-6 (IL-6), tumor necrosis factor-α (TNF-α), and interferon-γ (IFN-γ) in the lungs of the rPROS1-treated IAV/PR8-infected WT mice were also lower than those in the other groups ( Figure 3D). We also assessed lung damage via western blot analysis. Consistent with the reported resistance of alveolar type 1 (AT1) epithelial cells from C57BL/6 mice to IAV infection [30], the protein level of podoplanin, an AT1 epithelial cell marker, did not change during IAV infection ( Figure 3E,F). In contrast, the levels of pulmonary surfactant protein C (SP-C) and Clara cell 10-kDa protein (CC10), which are specific markers of AT2 and Clara epithelial cells, respectively, remained higher in the lungs of the rPROS1-treated IAV/PR8-infected WT mice than in those of the other infected groups, suggesting that AT2 and Clara cells are less damaged in the lungs under PROS1/AXL signaling ( Figure 3E,F). Compared with that in uninfected lungs, the level of the apoptotic marker cleaved caspase 3 was increased in IAV/PR8-infected lungs but was still much lower in the lungs of rPROS1-treated IAV/PR8-infected WT mice than in those of the other infected groups ( Figure 3E,F). The virus titers and levels of viral proteins, including PB1, NS1 and M2, were also lower in the lungs of the rPROS1-treated IAV/PR8-infected WT mice than in those of the other infected groups ( Figure 3G,H). Moreover, AXL ^–/–^ mice were more susceptible to IAV/PR8 infection, suggesting increased virus titers ( Figure 3G). These results indicated that PROS1/AXL signaling could suppress IAV infection-induced lung pathologies, including inflammatory cell infiltration, hemorrhage, hyaline membrane formation, inflammatory cytokine production, epithelial cell damage, and IAV production.

### PROS1/AXL recruits Gab1 and p85 to form a complex and activates PI3K/AKT/mTOR signaling

**Figure 4:**
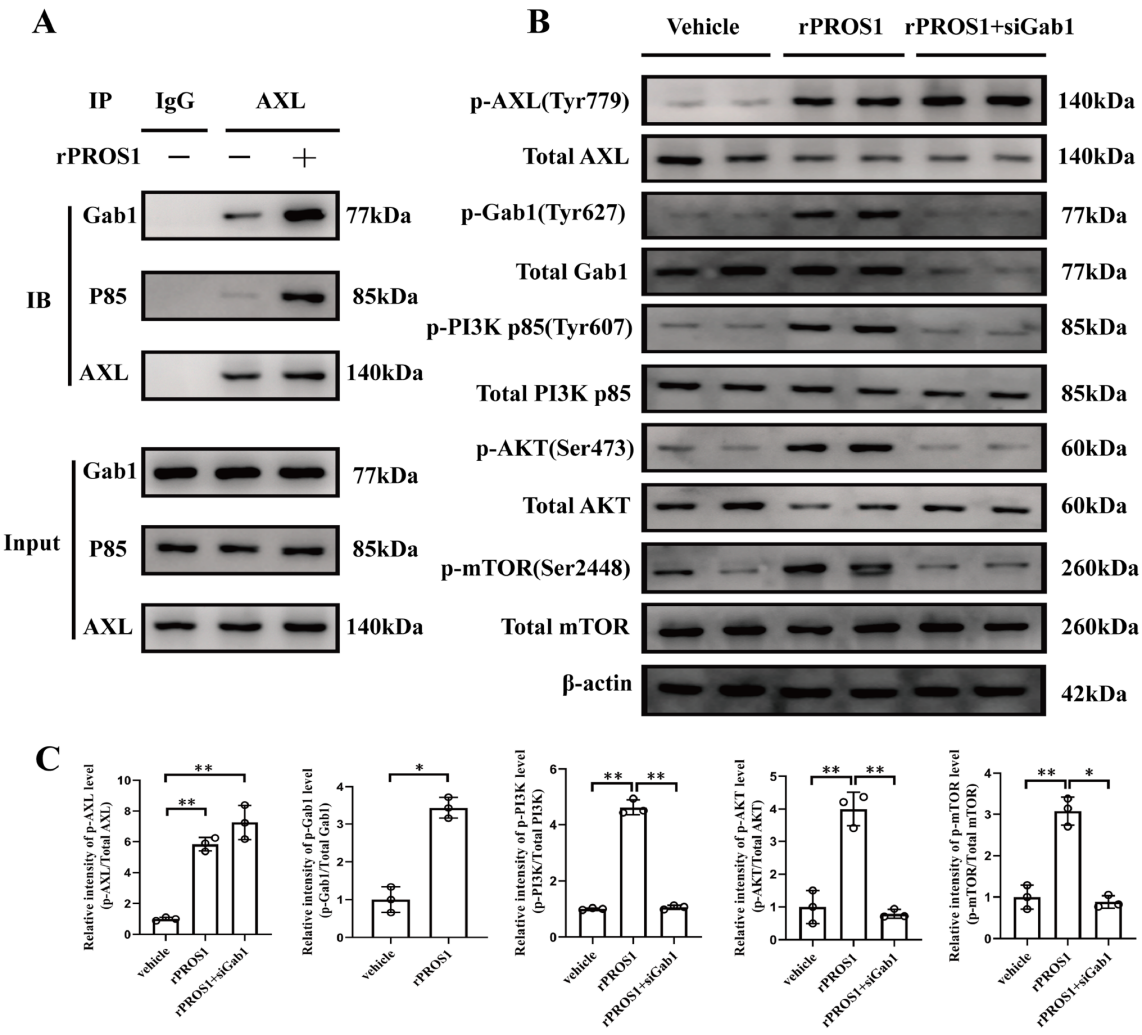
Figure 4 PROS1/AXL recruits Gab1 and p85 to form a complex and activates PI3K/AKT/mTOR signaling (A) Lung tissue lysates were prepared and used for an Coimmunoprecipitation assay with an anti-AXL antibody after treatment with rPROS1 (2 μg/mouse/day) for 4 days. Immunoblots (IBs) were performed with antibodies against Gab1, p85, and AXL. n = 3 for each group. (B) Western blot analysis revealed that rPROS1 (2 μg/mouse/day, for 4 days) induced the phosphorylation of AXL (Tyr779), Gab1 (Tyr627), PI3K p85 (Tyr607), AKT (Ser473), and mTOR (Ser2448) in lung tissue. Gab1 silencing via Gab1 siRNA blocked the PROS1-induced phosphorylation of Gab1, PI3K p85, AKT, and mTOR in lung tissue. n = 3 for each group. (C) Statistical plot of the relative density of phosphorylated proteins in the results (B) quantified by densitometric analysis. t-test for two-group comparisons. *P < 0.05 and **P < 0.01.

We used a database (BioGRID) to search for the presence of Gab1 in the list of predicted AXL interactors. In addition, tyrosine phosphorylation of Gab1 by flow shear stress could stimulate the association of Gab1 with the PI3K subunit p85 [31]. Therefore, we next questioned whether AXL could form a complex with Gab1/p85 upon rPROS1 treatment. We collected lung tissues from WT mice treated with vehicle buffer or rPROS1 and performed coimmunoprecipitation to detect complex formation with the indicated antibodies. The results revealed that AXL/Gab1/p85 complex formation increased upon stimulation with rPROS1 ( Figure 4A).

The PI3K/AKT signaling pathway has been reported to play a protective role in acute lung injury [ 32, 33] , and p85 is a regulatory subunit of PI3K; therefore, we explored whether the formation of the AXL/Gab1/p85 complex under rPROS1 stimulation could activate PI3K/AKT signaling and thus regulate the function of IAV-infected lungs. Western blot analysis results showed that rPROS1 induced the phosphorylation of AXL (at Tyr779), Gab1 (at Tyr627), p85 (at Tyr607), AKT (at Ser473), and mTOR (at Ser2448) in lung tissues ( Figure 4B,C). To determine whether rPROS1/AXL works through Gab1, we knocked down *Gab1* via specific siRNAs; the reduced expression of Gab1 blocked the PROS1/AXL signaling-induced phosphorylation of p85, AKT, and mTOR ( Figure 4B,C). These results suggest that PROS1/AXL can recruit Gab1 and p85 to form a complex and activate PI3K/AKT/mTOR signaling in the lungs.

### Activation of Gab1 and PI3K/AKT/mTOR signaling is essential for PROS1 to elicit protection against lethal IAV infection

**Figure 5:**
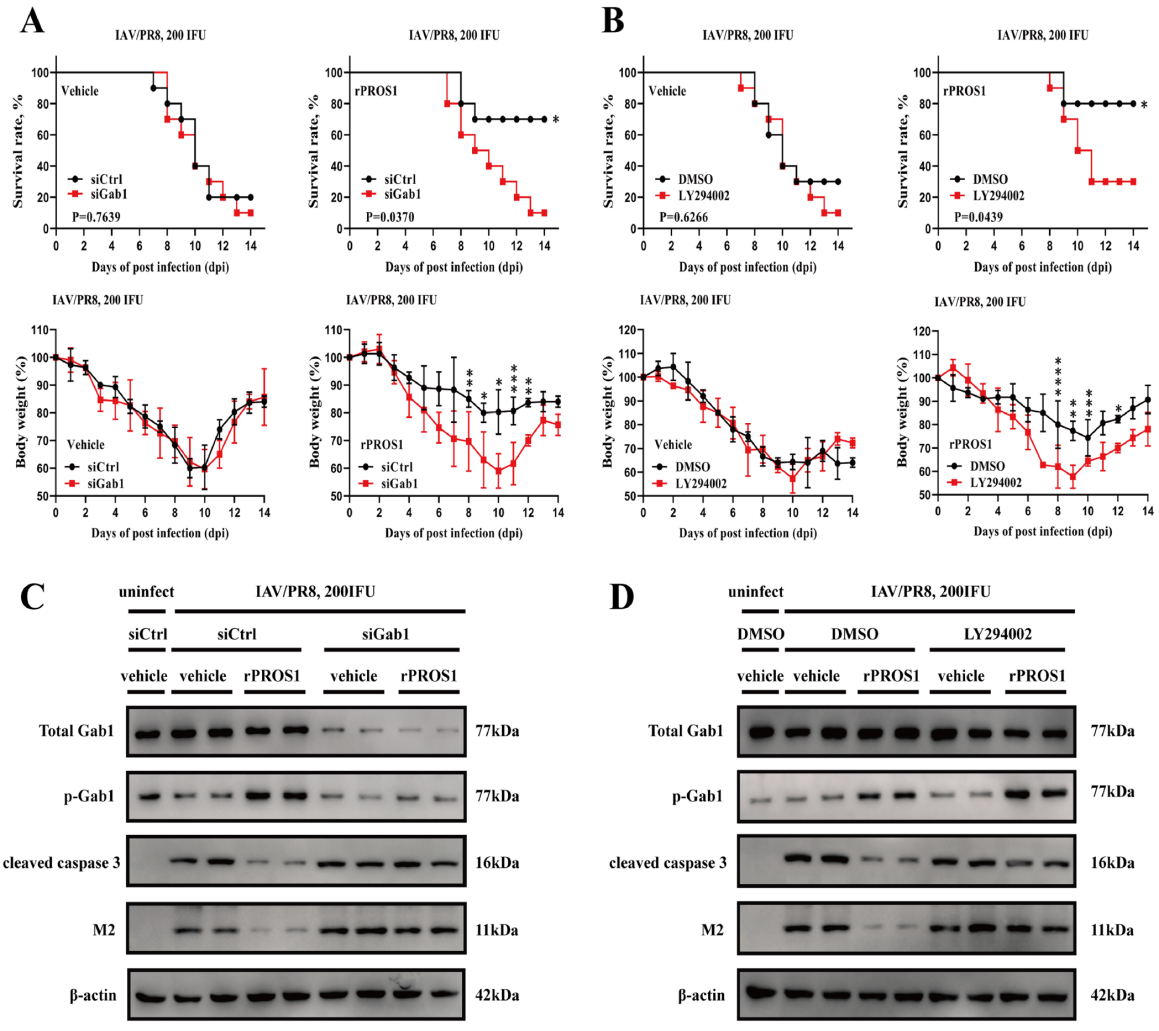
Figure 5 Activation of Gab1 and PI3K/AKT/mTOR signaling is essential for the ability of PROS1 to elicit protection against lethal IAV infection (A) Survival rate (%, upper panel) and body weight loss (%, lower panel) of WT mice transfected in vivo with siControl or siGab1 1 day before IAV/PR8 infection and then intranasally administered with either vehicle buffer alone (0 μg/mouse) or rPROS1 (2 μg/mouse) from day 1 to day 6 after transnasal infection with 200 IFU of IAV/PR8. n = 10 for each group. Error bars, SDs. (B) Survival rate (%, upper panel) and body weight loss (%, lower panel) of WT mice intranasally administered with vehicle buffer (0 μg/mouse) or rPROS1 (2 μg/mouse) alone or in combination with LY294002 (80 μg/mouse/day) from days 1 to 6 after transnasal infection with 200 IFU of IAV/PR8. n = 10 for each group. Error bars, SDs. (C) Western blot analysis of total Gab1, phosphorylated Gab1 (Tyr627), cleaved caspase 3, and the viral protein M2 in lungs from siCtrl/vehicle buffer-, siCtrl/rPROS1-, siGab1/vehicle buffer-, and siGab1/rPROS1-treated mice at 4 dpi with 200 IFU of IAV/PR8. Lungs from uninfected, siCtrl/vehicle buffer-treated mice were used as controls. n = 3 for each group. (D) Western blot analysis of total Gab1, phosphorylated Gab1 (Tyr627), cleaved caspase 3, and the viral protein M2 in lungs from DMSO/vehicle buffer-, DMSO/rPROS1-, LY294002/vehicle buffer-, and LY294002/rPROS1-treated mice at 4 dpi with 200 IFU of IAV/PR8. Lungs from uninfected, DMSO/vehicle buffer-treated mice were used as controls. n = 3 for each group. Survival rates were analyzed via the log-rank (Mantel-Cox) test. Body weight loss was analyzed via two-way ANOVA. *P < 0.05, **P < 0.01, ***P < 0.001 and ****P < 0.0001.

To determine whether the protective effect of PROS1/AXL against IAV infection-induced lethality is exerted through Gab1, we knocked down *Gab1* *in vivo* via specific siRNAs prior to IAV infection and then intranasally injected WT mice with vehicle buffer or rPROS1 (2 μg/per mouse/day) after transnasal infection with 200 IFU of IAV/PR8. Survival rate and weight loss analysis showed that in the vehicle group, 80%–90% of the infected mice died by 14 days, whereas rPROS1 significantly reduced mortality and weight loss in infected mice ( Figure 5A). However, silencing of *Gab1* drastically impaired the inhibitory effect of rPROS1 on mortality and weight loss in infected mice ( Figure 5A). Western blot analysis of mouse lung tissue also revealed that the expression of the apoptotic marker cleaved caspase 3 and the viral protein M2 was higher in the lungs of IAV/PR8-infected WT mice than in those of uninfected mice. rPROS1 treatment significantly reduced the levels of cleaved caspase 3 and the viral protein M2 in infected mice, but *Gab1* silencing inhibited the protective effect of rPROS1 ( Figure 5C).

Next, we investigated whether the protective effect of PROS1/AXL against IAV lethal infection occurs through the PI3K/AKT signaling pathway; we injected LY294002, an inhibitor of PI3K, transnasally after IAV infection. Similarly, compared with vehicle treatment, rPROS1 treatment significantly reduced mortality and weight loss in infected mice, but this protective effect of rPROS1 was significantly attenuated by LY294002 ( Figure 5B). Western blot analysis also revealed that IAV infection induced the expression levels of the cleaved caspase 3 and viral protein M2 were markedly reduced by rPROS1 treatment, but simultaneous treatment with LY294002 reversed the protective effect of rPROS1 ( Figure 5D). These data indicate that Gab1 and PI3K/AKT/mTOR signaling are essential for the ability of PROS1 to elicit protection against lethal IAV infection.

### PROS1/AXL activates Gab1 and the PI3K/AKT/mTOR pathway in macrophages in IAV-infected lungs

**Figure 6:**
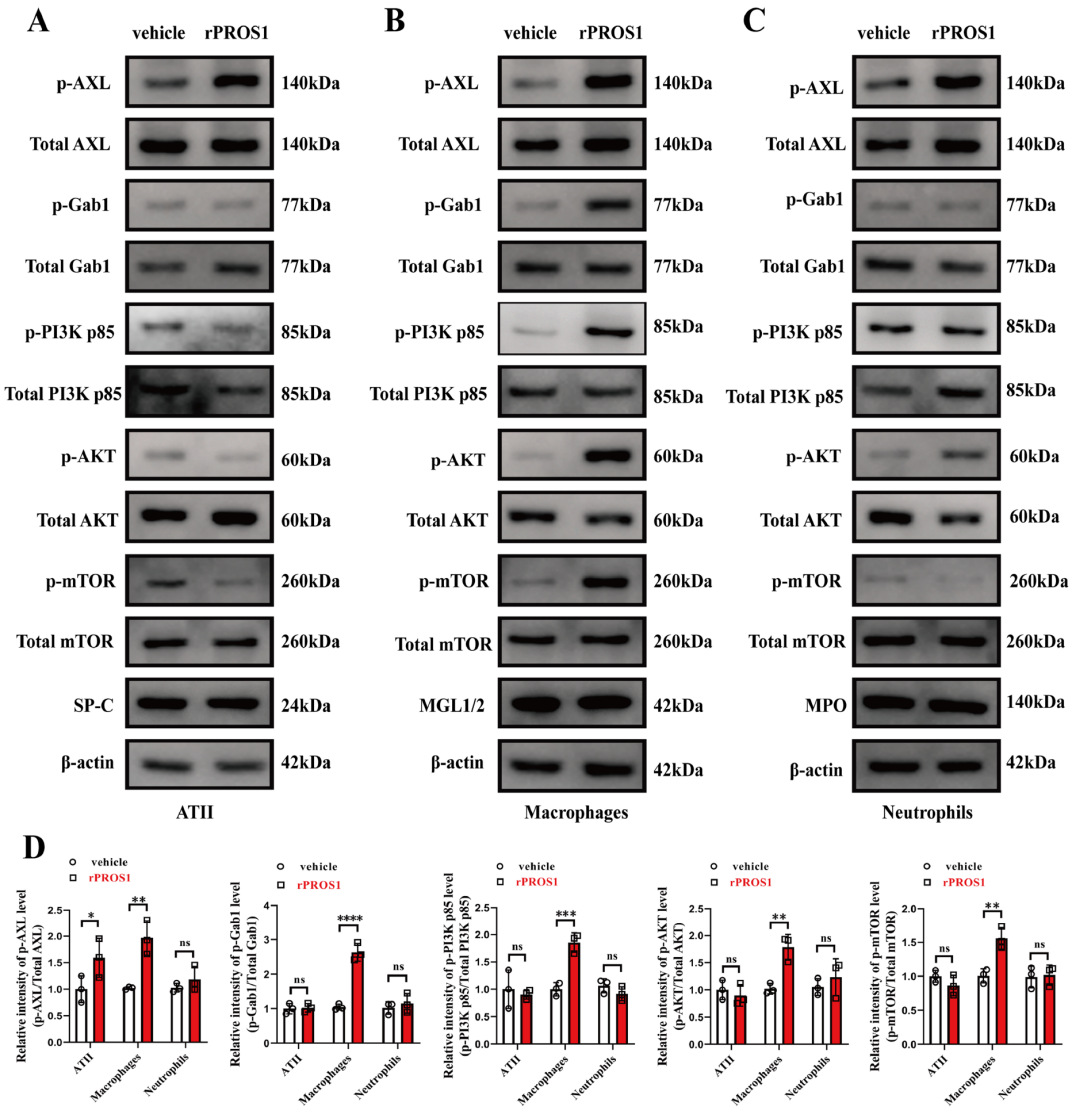
Figure 6 PROS1/AXL activates Gab1 and the PI3K/AKT/mTOR pathway in macrophages in IAV-infected lungs (A) Western blot analysis of the total and phosphorylated protein levels of AXL (Tyr779), Gab1 (Tyr627), PI3K p85 (Tyr607), AKT (Ser473), and mTOR (Ser2448), as well as the ATII cell marker SP-C, in ATII cells isolated from IAV/PR8-infected (200 IFU) and vehicle- or rPROS1-treated lungs (rPROS1, 2 μg/mouse/day for 4 days). n = 3 for each group. (B) Western blot analysis of the total and phosphorylated protein levels of AXL (Tyr779), Gab1 (Tyr627), PI3K p85 (Tyr607), AKT (Ser473), and mTOR (Ser2448) as well as the macrophage marker MGL1/2 in macrophages isolated from IAV/PR8-infected (200 IFU) and vehicle- or rPROS1-treated lungs (rPROS1, 2 μg/mouse/day, for 4 days). n = 3 for each group. (C) Western blot analysis of the total and phosphorylated protein levels of AXL (Tyr779), Gab1 (Tyr627), PI3K p85 (Tyr607), AKT (Ser473), and mTOR (Ser2448) as well as the neutrophil cell marker MPO in neutrophils isolated from IAV/PR8-infected (200 IFU) and vehicle- or rPROS1-treated lungs (rPROS1, 2 μg/mouse/day, for 4 days). n = 3 for each group. (D) Statistical plot of the relative density of phosphorylated proteins in the results (A,B,C) quantified by densitometric analysis. The data were analyzed via two-way ANOVA. *P < 0.05, **P < 0.01, ***P < 0.001 and ****P < 0.0001. ns, not significant.

AXL has been reported to be expressed in alveolar type II epithelial cells (ATII cells), alveolar macrophages, and neutrophils [ 7, 8] . To further elucidate the mechanism of rPROS1-induced protection against lethal IAV infection, we identified cell types in which AXL, downstream Gab1, and PI3K/AKT/mTOR were activated by rPROS1 in IAV/PR8-infected lungs. First, we isolated alveolar type II epithelial cells (ATII cells), alveolar macrophages, and alveolar neutrophils from IAV/PR8-infected and vehicle- or rPROS1-treated lungs. Next, we examined the total and phosphorylated protein levels of AXL, Gab1, PI3K, AKT, and mTOR in the isolated cells via western blot analysis. We also assayed various cellular markers to verify correct isolation, including pulmonary SP-C for ATII cells, macrophage galactose-type C-type lectins 1 and 2 (MGL1/2) for macrophages, and myeloperoxidase (MPO) for neutrophils. Western blot analysis results revealed that PROS1/AXL activated Gab1 and PI3K/AKT/mTOR only in macrophages in IAV-infected lung tissue ( Figure 6A–D).

### PROS1/AXL induces M2 polarization through activation of Gab1 in alveolar macrophages

**Figure 7:**
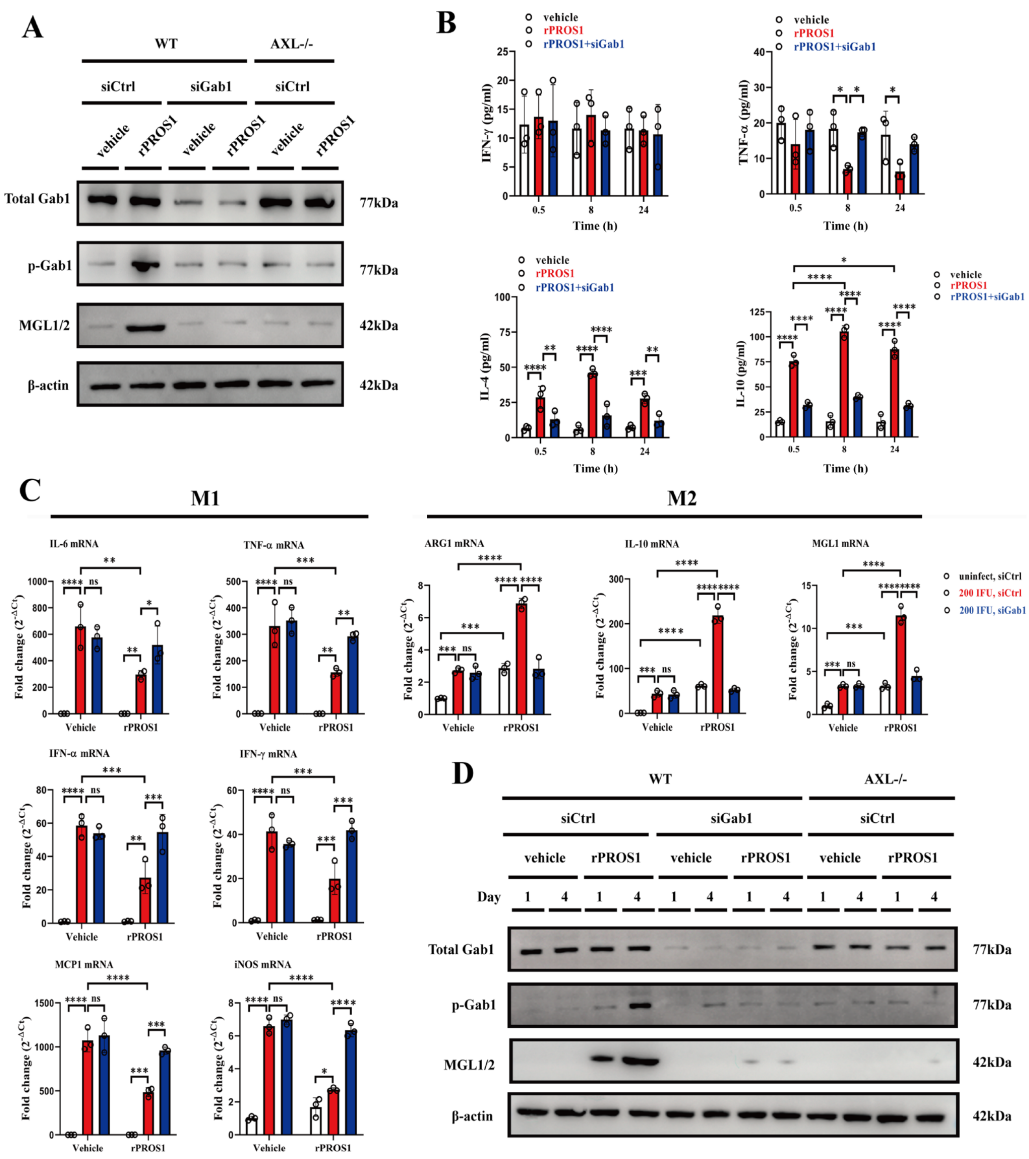
Figure 7 PROS1/AXL induces M2 polarization through the activation of Gab1 in alveolar macrophages (A) Western blot analysis of total Gab1, phosphorylated Gab1 (Tyr627), and MGL1/2 in alveolar macrophages from WT and AXL–/– mice 3 hours after treatment with vehicle buffer or rPROS1 together with siControl or siGab1. n = 3 for each group. (B) ELISA for IFN-γ, TNF-α, IL-4, and IL-10 in the culture medium of WT alveolar macrophages at 0.5, 8, and 24 h after treatment with vehicle buffer, rPROS1, and rPROS1/siGab1. n = 3 for each group. (C) Fold mRNA expression (2–∆Ct) analyzed by real-time PCR for IL-6, TNF-α, IFN-α, IFN-γ, MCP1, iNOS, ARG1, IL-10, and MGL1 in the lungs of vehicle buffer/siCtrl-, vehicle buffer/siGab1-, rPROS1/siCtrl-, and rPROS1/siGab1-treated mice uninfected and at 4 dpi with 200 IFU of IAV/PR8. n = 3 for each group. (D) Western blot analysis of total Gab1, phosphorylated Gab1 (Tyr627), and MGL1/2 in alveolar macrophages from WT and AXL–/– mice 1 and 4 days after intranasal administration of vehicle buffer or rPROS1 (2 μg/mouse/day) together with control siRNA or Gab1 siRNA in vivo.n = 3 for each group. The data were analyzed via two-way ANOVA. *P < 0.05, **P < 0.01, ***P < 0.001 and ****P < 0.0001. ns, not significant.

MGL1/2 is a specific marker for M2 macrophages, and the mTOR-dependent signaling pathway can stimulate the M2 polarization of macrophages [34]. Therefore, we further clarified the role of the PROS1/AXL-induced activation of Gab1 in macrophage polarization. First, we collected alveolar macrophages from uninfected WT or AXL ^–/–^ mice and incubated them with rPROS1 *in vitro*. As shown in western blot analysis, incubation with rPROS1 increased the levels of phosphorylated Gab1 (at Tyr627) and MGL1/2 in WT macrophages but not in AXL ^–/–^ macrophages ( Figure 7A). Gab1 activation and MGL1/2 upregulation in rPROS1-treated macrophages were suppressed by *Gab1* silencing ( Figure 7A). ELISA results showed that the M2 cytokines IL-4 and IL-10, but not the M1 cytokines IFN-γ and TNF-α, were increasingly released into the medium from rPROS1-treated WT macrophages but were inhibited by *Gab1* silencing ( Figure 7B). We further evaluated the M1/M2 macrophage status in rPROS1-treated, IAV/PR8-infected lungs by detecting the expression levels of M1 and M2 macrophage-specific genes via real-time PCR. The expression levels of M1-specific genes, such as those encoding IL-6, TNF-α, IFN-α, IFN-γ, monocyte chemotactic protein-1 (MCP-1) and inducible nitric oxide synthetase (iNOS), were lower in the rPROS-treated, IAV/PR8-infected lungs than in the vehicle buffer-treated, IAV/PR8-infected lungs ( Figure 7C). In contrast, the expression of M2-specific genes, including those encoding arginase 1 (ARG1), MGL1, and IL-10, was upregulated in rPROS1-treated, IAV/PR8-infected lungs ( Figure 7C). In addition, *Gab1* silencing increased the expression of M1-specific genes and decreased the expression of M2-specific genes in rPROS1-treated, IAV/PR8-infected lungs ( Figure 7C). These data suggested that the rPROS1-induced activation of Gab1 in macrophages could lead to macrophage polarization toward the M2 phenotype in IAV-infected lungs. We also silenced or did not silence *Gab1* *in vivo* with specific Gab1 siRNA or control siRNA and then injected rPROS1 and vehicle buffer directly into the nasal cavities of WT mice or AXL ^–/–^ mice and collected alveolar macrophages 1 and 4 days after injection. Western blot analysis revealed that WT alveolar macrophages, but not AXL ^–/–^ alveolar macrophages, presented increased levels of phosphorylated Gab1 (at Tyr627) and MGL1/2 after treatment with rPROS1 ( Figure 7D). The increase in phosphorylated Gab1 (at Tyr627) and MGL1/2 in rPROS1-treated WT mice was abolished by *Gab1* silencing ( Figure 7D). These results suggested that PROS1/AXL signaling could lead to the activation of Gab1 and downstream PI3K/AKT/mTOR in macrophages and polarize them to M2 macrophages, which reduces inflammatory injury mediated by IAV infection in the lungs.

### PROS1 also protects against lethal infection with other IAV strains

**Figure 8:**
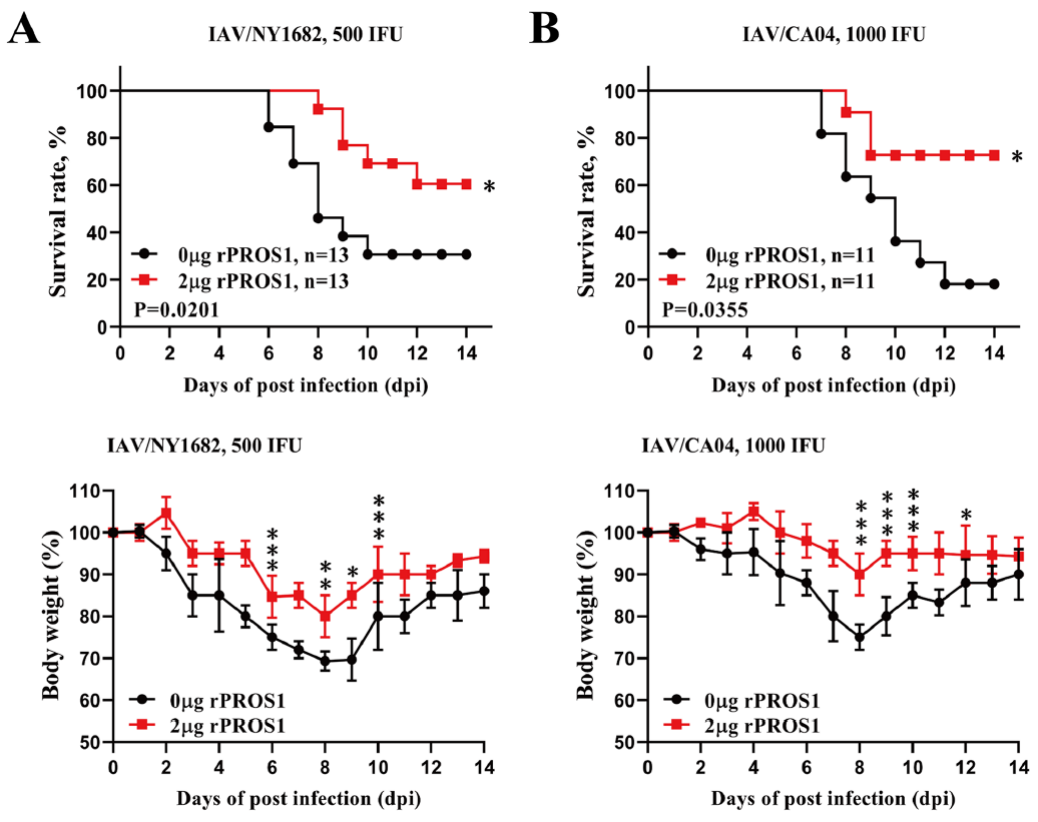
Figure 8 PROS1 also protects against lethal infection with other IAV strains Survival rate (%, upper panels) and body weight loss (%, lower panels) of WT mice intranasally administered with vehicle buffer alone (0 μg/mouse) or rPROS1 (2 μg/mouse) from day 1 to day 6 after transnasal infection with 500 IFU of IAV/NY1682 (n = 13 for each group) (A) or 1000 IFU of IAV/CA04 (n = 11 for each group) (B). Error bars, SDs. Survival rates were analyzed via the log-rank (Mantel-Cox) test. Body weight loss was analyzed via two-way ANOVA. *P < 0.05, **P < 0.01, and ***P < 0.001.

We further investigated whether PROS1 could protect against other IAV strains by intranasally administering PROS1 (2 μg/per mouse/day) to WT mice from day 1 to day 6 after transnasal infection with 500 IFU of IAV/NY1682 and 1000 IFU of IAV/CA04. Similarly, control vehicle buffer was injected into WT mice. The mortality rates of the control mice infected with IAV/NY1682 and IAV/CA04 were approximately 70% and 80%, respectively ( Figure 8A,B). However, PROS1 treatment significantly reduced mortality to approximately 40% and 30% in mice infected with IAV/NY1682 and IAV/CA04, respectively ( Figure 8A,B). Weight loss was also reduced in infected mice by the treatment with PROS1 ( Figure 8A,B). These results indicated that PROS1 could confer protection against lethal infection with various IAV strains.

## Discussion

In the present study, we showed that treatment with recombinant PROS1 (rPROS1) conferred protection against lethal infection with IAVs in WT mice. rPROS1 mitigated inflammatory cell infiltration, inflammatory cytokine production, epithelial cell damage and virus production in IAV-infected lungs and ultimately reduced the morbidity and mortality of IAV-infected mice. We also observed that treatment with rPROS1 suppressed caspase 3 activity in IAV-infected lungs, indicating that rPROS1 is not toxic to cells. However, rPROS1 failed to protect AXL ^–/–^ mice from lethal infection with IAV/PR8, confirming that targeting AXL with PROS1 is essential to produce protective activity against IAV infection. Mechanistically, PROS1 induces the phosphorylation of AXL, and activated AXL recruits Gab1 and p85, a regulatory subunit of PI3K, to form a complex that activates Gab1 and its downstream PI3K/AKT/mTOR in macrophages and promotes their polarization to M2, which ultimately inhibits IAV infection-induced inflammatory damage in the lungs and mortality in mice. Overall, PROS1/AXL signaling and its facilitation of M2 macrophage polarization constitute novel therapeutic targets for IAV infection.

In mouse lungs, the mRNA and protein expression of TAM receptors is detected in alveolar type II epithelial cells (ATII cells), alveolar macrophages and neutrophils [ 7– 10] . The expression and secretion of Gas6 and PROS1 have also been detected in mouse lungs and BALF [ 9, 17] . Consistently, we detected basal expression of TAM receptors and their ligands in the lungs and BALF of mice, where the expression of AXL, PROS1 and Gas6 was elevated after IAV/PR8 infection, whereas the expression of Tyro3 and Mertk was unchanged, suggesting that AXL and its ligands may play important functions in IAV infection. Exogenous administration of PROS1 or Gas6 alleviated acute lung injury in mice by inhibiting inflammatory cytokine secretion through the activation of AXL [ 16, 17] . The AXL agonist Gas6 binds to phosphatidylserine exposed on the membrane surface of apoptotic cells, thereby bridging apoptotic cells to phagocytes expressing AXL [ 35, 36] . Defects in efferocytosis due to AXL deficiency and the consequent failure of inflammation resolution are associated with the onset/progression of a variety of chronic lung inflammatory disorders [37]. Reports have also shown that loss of AXL results in a reduced ability to initiate an adequate adaptive antiviral response, as exemplified by deficient priming of T cells after IAV and MNV infection [18]. Consistently, our study revealed that PROS1, but not Gas6, attenuated inflammatory damage and virus production in IAV-infected lungs through the activation of AXL and ultimately reduced the mortality of IAV-infected mice, whereas AXL ^–/–^ mice were more susceptible to IAV infection. However, a paradox also exists in that AXL receptor blockade ameliorates virus-induced lung pathology as well as drug-induced pulmonary fibrosis [ 9, 10] ; therefore, an in-depth study of the function of AXL and its ligands in distinct viral infections as well as the underlying mechanisms is indispensable.

Gab1 is known to serve as an adaptor protein that integrates signals from different receptors and affects signaling cascades such as the PI3K/AKT and MAPK/ERK cascades [ 38, 39] . The function of Gab1 is poorly understood, and it has been reported to protect against septic lung injury [ 40, 41] . By revealing the intrinsic mechanism, we found that PROS1 induced AXL phosphorylation and that activated AXL recruited Gab1 and p85, a regulatory subunit of PI3K, to form a complex that activated Gab1 and its downstream PI3K/AKT/mTOR. Furthermore, the PI3K/AKT pathway also confers protection against acute lung injury [ 32, 33] . Therefore, we investigated whether the protective effect of PROS1 against IAV-induced lethal infections occurs through the AXL/Gab1/PI3K/AKT pathway. We found that Gab1 siRNA and LY294002, which inhibit Gab1 as well as PI3K, abolished the PROS1-induced protective activity against IAV infection, suggesting that the PROS1/AXL-induced activation of Gab1 and PI3K/AKT is essential for the PROS1-induced protective activity against IAV infection. Administration of Gab1 siRNA or LY294002 alone did not affect the mortality of IAV/PR8-infected mice, suggesting that the Gab1 or PI3K/AKT pathway is not critically involved in the pathogenesis of IAV infection.

Owing to the characteristic distribution of AXL in the lung, we isolated different cells, such as alveolar type II epithelial cells, alveolar macrophages, and alveolar neutrophils, and found that the PROS1/AXL-induced activation of Gab1 and the PI3K/AKT/mTOR pathway occurred predominantly in alveolar macrophages and that their activation may affect the function of alveolar macrophages. Macrophages are phagocytic cells that activate innate immunity and modulate the inflammatory response [ 42, 43] . In terms of phenotype and function, macrophages can be classified into two types: the proinflammatory M1 type and the anti-inflammatory M2 type [ 42, 43] . In the early stages of infection, macrophages are polarized into the M1 type, which activates innate immunity through the secretion of inflammatory cytokines such as IL-1, IL-6 and TNF-α, thus reducing pathogen infection. In contrast, in the late stages of infection, macrophages are polarized into the M2 type, which reduces the inflammatory response and removes damaged cells by secreting anti-inflammatory cytokines such as TGF-β and IL-10 [ 42, 43] . Several studies have demonstrated that M2 macrophages could be therapeutically beneficial for IAV infection [ 44, 45] . Furthermore, the PROS1 and PI3K/AKT/mTOR pathways can favor macrophage polarization into the M2 type [ 20, 46, 47] . It is thus likely that PROS1/AXL-induced M2 macrophage polarization through the activation of Gab1 and PI3K/AKT/mTOR is the underlying mechanism for the PROS1-induced protection against IAV infection.

We found that Gab1 was predominantly activated in M2 macrophages in PROS1-treated, IAV-infected lungs and that Gab1 siRNA reduced the number of M2 macrophages and conversely increased the number of M1 macrophages in PROS1-treated, IAV-infected lungs. We also found that PROS1 activated Gab1 in alveolar macrophages from WT mice but not in those from AXL ^–/–^ mice and induced M2 polarization in WT macrophages *in vitro* and that Gab1 siRNA inhibited PROS1-induced M2 polarization in WT macrophages. We further showed that PROS1 activated Gab1 in alveolar macrophages and induced M2 macrophage polarization *in vivo*. These results suggest that PROS1 could activate Gab1 through interaction with the AXL receptor on the cell surface of macrophages and induce M2 macrophage polarization. Alveolar macrophages incubated with PROS1 secrete large amounts of IL-4 and IL-10 into the culture medium; these cytokines are known to induce M2 macrophage polarization via the STAT6 pathway [42]. Therefore, it is possible that IL-4 and IL-10 are involved in the PROS1-induced polarization of M2 macrophages.

Together, we showed that targeting AXL with PROS1 could induce M2 macrophage polarization through the activation of Gab1 and downstream PI3K/AKT/mTOR signals, thereby conferring protection against lethal influenza infection in mice. These results revealed a novel function of the PROS1/AXL signal in M2 macrophage polarization and suggested that PROS1/AXL-induced M2 macrophage polarization could be a novel therapeutic target for influenza infection. However, it is also important to maintain caution as to whether PROS1 has adverse effects in forcing macrophages to polarize to the M2 type.

## Supporting information

25413FigS1-TabS1-2

## Supplementary Data

Supplementary data are available at *Acta Biochimica et Biophysica Sinica* online.
