# Supplementary material for: PROS1/AXL signaling protects mice from lethal influenza infection by inducing M2 macrophage polarization: The PROS1/AXL axis protects mice from lethal influenza infection
Source: Acta Biochim Biophys Sin (Shanghai). 2025 Oct 29;58(5):1055–68. doi: 10.3724/abbs.2025169 (PMC13191473; doi:10.3724/abbs.2025169)
Supplement: 25413FigS1-TabS1-2 [file 25413FigS1-TabS1-2.doc]

**
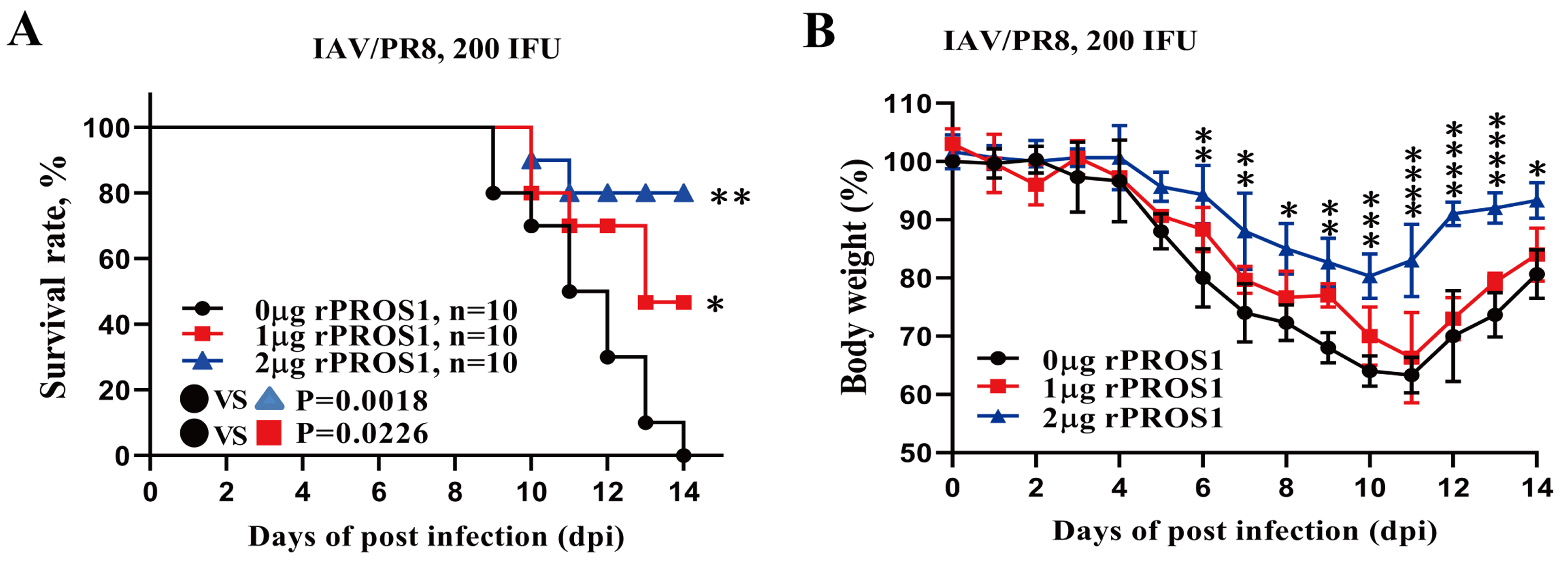
**

**Supplementary Figure S1. rPROS1 treatment reduces the mortality and weight loss of IAV/PR8-infected mice in a dose-dependent manner** The survival rate (%, upper panel) and body weight loss (%, lower panel) of WT mice intranasally administered with either the vehicle buffer alone (0µg/mouse) and 1µg/mouse and 2µg/mouse of rPROS1 from day 1 to day 6 after transnasal infection with 200 IFU of IAV/PR8. *n*=10 for each group. Error bars, SD. Survival rates were analyzed using the log-rank (Mantel-Cox) test. Body weight loss were analyzed using two-way ANOVA. **P*<0.05, ***P*<0.01, ****P*<0.001 and *****P*<0.0001.

**Supplementary Table S1.** **Chemical reagents, commercial kits and antibodies used in this study**

| **Chemical reagents** | **Corporation** | **Catalog number** | **Working concentration** |
| --- | --- | --- | --- |
| Recombinant Gas6 | Abnova | cat. H00002621-P01 | 200µg/ml for mice |
| rPROS1 | Abnova | cat. H00005627-P01 | 200µg/ml for mice |
| LY294002 | Sigma | cat. 440202 | 80µg/50µl in saline with 0.1% DMSO |

|  | | |
| --- | --- | --- |
| **Commercial kits** | **Corporation** | **Catalog number** |
| Gas6 ELISA kit | Abcam | cat.ab155447 |
| PROS1 ELISA kit | Zci Bio | cat.ZC-57259 |
| BCA protein Assay kit | CWBIO | cat.CW0014S |
| IL-6 ELISA kit | R&D systems | cat.M6000B |
| TNF-α ELISA kit | R&D systems | cat.MTA00B |
| IFN-γ ELISA kit | R&D systems | cat.MIF00 |
| IL-4 ELISA kit | R&D systems | cat.M4000B |
| IL-10 ELISA kit | R&D systems | cat.M1000B |
|  | | |
| **Antibodies** | **Corporation** | **Catalog number** |
| anti-AXL | Abcam | cat.ab215205 |
| anti-Tyro3 | R&D Systems | cat.AF759 |
| anti-Mertk | Abcam | cat.ab300136 |
| anti-Podoplanin | R&D Systems | cat.AF3244 |
| anti-SP-C | Santa Cruz Biotechnology | cat.sc-518029 |
| anti-CC10 | Santa Cruz Biotechnology | cat.sc-390313 |
| anti-pro-caspase 3 | Cell signaling | cat.9662S |
| anti-cleaved caspase 3 | Cell signaling | cat.9664S |
| anti-β-actin | Proteintech | cat.66009-1-Ig |
| anti-PB | GeneTex | cat.GTX125923 |
| anti-NS1 | GeneTex | cat.GTX125990 |
| anti-M2 | GeneTex | cat.GTX125951 |
| anti-NP | GeneTex | cat.GTX125989 |
| anti-AXL IP antibody | Abcam | cat.ab314097 |
| anti-phospho-AXL (Tyr779) | R&D Systems | cat.AF2228 |
| anti-Gab1 | Abcam | cat. ab59362 |
| anti-phospho-Gab1 (Tyr627) | Invitrogen | cat.PA5-36846 |
| anti-PI3 Kinase p85 | Cell signaling | cat.4292 |
| anti-phospho-PI3K p85 alpha (Tyr607) | Invitrogen | cat.PA5-104853 |
| anti-AKT | Cell signaling | cat.4691S |
| anti-phospho-AKT (Ser473) | Cell signaling | cat.4060S |
| anti-mTOR | Cell signaling | cat.2983T |
| anti-phospho-mTOR (Ser2448) | Cell signaling | cat.5536T |
| anti-MGL1/2 | R&D Systems | cat. AF4297 |
| anti-MPO | Proteintech | cat.66177-1-Ig |
| HRP-conjugated anti-goat IgG antibody | R&D Systems | cat.HAF017 |
| HRP-conjugated anti-rabbit IgG antibody | Cell signaling | cat.7074 |
| HRP-conjugated anti-mouse IgG antibody | Cell signaling | cat.7076 |

Note: The dilution ratio of all primary antibodies is 1:1000, and that of all secondary antibodies is 1:5000.

**Supplementary Table S2. Sequences of** primers used in this study

| Genes(Mouse) | Primers | Sequence (5′-3′) |
| --- | --- | --- |
| *GAPDH* | Forward | AGCCTCAAGATCATCAGCAATGCC |
|  | Reverse | TGTGGTCATGAGTCCTTCCACGAT |
| *Gas6* | Forward | TGCTGGCTTCCGAGTCTTC |
|  | Reverse | CGGGGTCGTTCTCGAACAC |
| *PROS1* | Forward | CCAAGCTGCTTTTACTTGCTTC |
|  | Reverse | GCCTCCATTGACATTTGAGGG |
| *AXL* | Forward | GGAACCCAGGGAATATCACAGG |
|  | Reverse | AGTTCTAGGATCTGTCCATCTCG |
| *Tyro3* | Forward | AGATGACCGTGTCTCAGGGG |
|  | Reverse | ACCGTTCCACTGACTTTAGGC |
| *Mertk* | Forward | CTCCTGAGCCCGTCAATATCT |
|  | Reverse | AGACCAGGTACGGTTAGGACA |
| *IL-6* | Forward | CGGCCTTCCCTACTTCACAA |
|  | Reverse | GGATGGTCTTGGTCCTTAGC |
| *TNF-α* | Forward | GCCTATGTCTCAGCCTCTTC |
|  | Reverse | GGAGGTTGACTTTCTCCTGG |
| *IFN-α* | Forward | TGGCTAGGCTCTGTGCTTTC |
|  | Reverse | AGCTGCTGGTGGAGGTCATT |
| *IFN-γ* | Forward | CTTGGCTTTGCA GCTCTTCC |
|  | Reverse | |  | | --- |   GCTCATTGAATGCTTGGCGC |
| *MCP1* | Forward | CCTGCTGTTCACAGTTGC |
|  | Reverse | GTCTGGACCCATTCCTTC |
| *iNOS* | Forward | CCAAGCCCTCACCTACTTCC |
|  | Reverse | CACTTCGCACAAAGCAGGGC |
| *ARG1* | Forward | TGGCTTGCGAGACGTAGAC |
|  | Reverse | CTCCTCTGCTGTCTTCCCA |
| *IL-10* | Forward | ATACTGCTAACCGACTCCT |
|  | Reverse | ATGGCCTTGTAGACACCT |
| *MGL1* | Forward | GCTTCGAAAAAGGGATCAGTTCT |
|  | Reverse | CCCAGTTCTTAAAGCCTTTCTCA |
|  |  |  |
